# Technique integration of single-cell RNA sequencing with spatially resolved transcriptomics in the tumor microenvironment

**DOI:** 10.1186/s12935-022-02580-4

**Published:** 2022-04-19

**Authors:** Hailan Yan, Jinghua Shi, Yi Dai, Xiaoyan Li, Yushi Wu, Jing Zhang, Zhiyue Gu, Chenyu Zhang, Jinhua Leng

**Affiliations:** 1 Department of Obstetrics and Gynecology, Peking Union Medical College Hospital, Chinese Academy of Medical Sciences & Peking Union Medical College, No.1 Shuaifuyuan Dongcheng District, Beijing, 100730 China; 2 National Clinical Research Center for Obstetric & Gynecologic Diseases, No.1 Shuaifuyuan Dongcheng District, Beijing, 100730 China

**Keywords:** Integration, Single-cell RNA sequencing, Spatially resolved transcriptomics, Tumor microenvironment

## Abstract

**Background:**

The tumor microenvironment contributes to tumor initiation, growth, invasion, and metastasis. The tumor microenvironment is heterogeneous in cellular and acellular components, particularly structural features and their gene expression at the inter-and intra-tumor levels.

**Main text:**

Single-cell RNA sequencing profiles single-cell transcriptomes to reveal cell proportions and trajectories while spatial information is lacking. Spatially resolved transcriptomics redeems this lack with limited coverage or depth of transcripts. Hence, the integration of single-cell RNA sequencing and spatial data makes the best use of their strengths, having insights into exploring diverse tissue architectures and interactions in a complicated network. We review applications of integrating the two methods, especially in cellular components in the tumor microenvironment, showing each role in cancer initiation and progression, which provides clinical relevance in prognosis, optimal treatment, and potential therapeutic targets.

**Conclusion:**

The integration of two approaches may break the bottlenecks in the spatial resolution of neighboring cell subpopulations in cancer, and help to describe the signaling circuitry about the intercommunication and its exact mechanisms in producing different types and malignant stages of tumors.

## Background

Cancer was viewed as a heterogeneous disease with a succession of genetic changes which led to the conversion of normal cells into malignant cells [1]. In 1889, Paget first came up with the theory of “seed and soil,” postulating the relationship between tumor and tumor microenvironment (TME) [2]. TME comprises cellular and acellular components such as stromal cells, myeloid cells, lymphoid cells, and extracellular matrix (ECM). Now it’s evident that TME plays an essential role in tumorigenesis, having diverse capacities to induce both beneficial and adverse consequences in tumor initiation, growth, invasion, and metastasis [3, 4]. However, there are still many unexplored questions in this field. The emerging problem is how to explore and manage TME diversity, given that both structural features and gene expression in TME are particularly heterogeneous at the inter-and intra-tumor levels [4, 5]. Interactions like intercellular communication need to be investigated forward, as TME is a complex, spatially restricted network [6, 7].

Technological developments have advanced our understanding of tumor biology. Many researchers have utilized RNA-sequencing (RNA-seq) based on next-generation sequencing (NGS) to measure tissue transcriptomes [8, 9]. Traditionally, bulk RNA-sequencing (bulk RNA-seq) is widely used to sequence a mixture of RNA transcripts from the whole tissue profiling averages of cellular expression [10]. Nevertheless, it has lost information about cellular heterogeneity. Single-cell RNA sequencing (scRNA-seq) improves and makes it possible to profile the transcriptome of single cells and infer cell type and trajectory [11, 12]. Whereas, scRNA-seq has failed to acquire spatial information, which is critical to understanding the functionality and pathological changes of tissues that are dissociated in suspension [9, 12, 13]. In addition, spatially resolved transcriptomics (SRT) has been developed to reveal spatial information and study spatial heterogeneity with the drawbacks of coverage or depth of transcripts. Computational developments have enabled the combination of scRNA-seq and spatial transcriptomics data to get through their limitations and make use of their favorable factors.

## Developments and limitations of SRT

SRT technologies can be divided into four categories: technologies based on microdissected gene expression, in situ hybridization (ISH) technologies, in situ sequencing (ISS) technologies, and in situ capturing technologies [14].

The typical microdissected method is laser capture microdissection (LCM), which cuts out tissue regions precisely and isolates specific, pure cells from their heterogeneous environments by a laser beam under a microscope [15]. LCM sequencing (LCM-seq) combining LCM with RNA-seq profiles gene expression of selected tissue regions, elucidating cellular heterogeneity and spatial variance with low throughput and requirements in a large number of cells [16]. Geo-seq using scRNA-seq coupled with LCM advances the analysis at a resolution of as few as 10 cells. Yet, it’s still laborious and can’t attain single-cell resolution [17].

ISH technologies are early attempts to visualize gene expression in fixed tissue, as exemplified by single-molecule RNA fluorescence in situ hybridization (smFISH). Many short oligonucleotide probes labeled with fluorophores are hybridized to different regions of the same mRNA transcript [18–20]. SmFISH has high sensitivity and subcellular spatial resolution but a low target throughput of around 1–4 transcripts and up to 100 cells per handle [18, 19, 21, 22]. ISH technologies also include multiplexed error-robust fluorescence in situ hybridization (MERFISH), sequential fluorescence in situ hybridization (seqFISH), and ouroboros smFISH (osmFISH).

ISS technologies are methods for parallel targeted analysis of short RNA fragments in morphologically preserved cells and tissue [23]. ISS technologies encompass ISS using padlock probes, fluorescent in site RNA sequencing (FISSEQ), and spatially resolved transcript amplicon readout mapping (STARmap) [14]. ISS using padlock probes is the first ISS approach that can detect single nucleotide variants (SNV) compared to ISH. In human breast cancers, ISS detected targeted mRNAs and measured 31 genes at a subcellular spatial resolution of about 450 cells per single handle [23]. Some studies have concluded that ISH and ISS technologies are image-based in situ transcriptomics because they are all targeted in situ methods using probes to represent quantitative RNA analysis characterized by great depth and low coverage [11]. The principle of in situ capturing technologies is to capture transcripts in situ, then sequence them ex-situ.

Ståhl et al. first proposed spatial transcriptomics (ST), depositing a customized slide with a diameter of 100 μm microarray features over an area of 6.2 mm by 6.6 mm to capture transcripts. There are over 200 million oligonucleotides used to capture mRNAs in each of the 1007 features. Each microarray feature contains unique DNA-barcoded probes including a cleavage site, a T7 amplification and sequencing handle, a spatial barcode, a unique molecular identifier (UMI), and an oligo (dT) VN-capture region. After capturing and reverse-transcribing mRNA, cDNA synthesis from tissue with arrayed oligonucleotides on a surface is carried out. Then, RNA-seq is used to image gene expression while maintaining positional information [9]. Although this method could provide spatially resolved whole-transcriptome information and be more accessible, its limitations in resolution and depth can’t be ignored.

## Integration approaches of scRNA-seq and SRT

Each current method of SRT mentioned above has its strengths and drawbacks. Hence, to meet the demands of exploring spatial patterning of gene expression at a single cell even subcellular resolution in an intricate environment, it’s necessary to integrate scRNA-seq and SRT to maximize the benefits. Two approaches currently exist for the integration of non-spatial scRNA-seq and SRT: (i) experimental improvement strategies, (ii) computing strategies.

One of the experimental improvement strategies called XYZeq improves experiments by using two rounds of split-pool barcoding to encode the spatial information at single-cell resolution from a sample into scRNA-seq [24–26]. ST is applied to a tissue sample in the first round aiming to get positional information. The innovative step in the second round is to remove intact cells from microarrays, pool them, and amplify indexing with a combinatorial barcode per single cell and sequence. This method maps a single cell’s physical location in the array by spatial barcode [26].

**Fig. 1:**
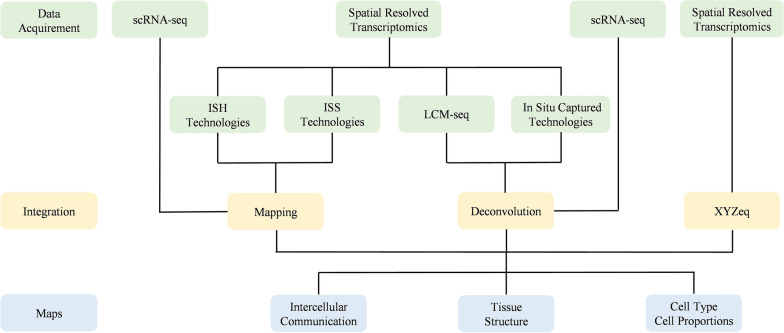
A workflow for integration

Advances in computing techniques have made copy number inference and fusion transcription recognition possible [27]. Mapping and deconvolution are two key steps to achieving this goal. The mapping process is to distribute cell types and states based on scRNA-seq to each cell spatially resolved by image-based in situ transcriptomics. It also predicts the locations of spatially confined and dispersed subpopulations [10, 28, 29]. Deconvolution is the process of estimating cell-type proportions in spatial data at a microdissected or in situ capturing spot, informed by single-cell data [30]. Here is a workflow for integration (Fig. 1). The first step is to establish discrete cell subtypes through scRNA-seq and investigate tissue structures of interest from spatial data, which comes from the same or different biopsy as well as reference databases. Then, mapping or deconvolution as in silico strategies is applied to scRNA-seq and SRT data to understand the architecture of the cell-type distribution and the putative mechanisms of intercellular communication based on this architecture [10]. Computing strategies have overcome drawbacks of each technology and provided spatially resolved whole-transcriptome information at single-cell resolution yielding greater coverage and depth. Mapping could create spatially resolved maps at single-cell resolution. Deconvolution strategies enable estimating cell-type proportions and characterizing specific gene expression and other biological processes. However, the integration of scRNA-seq and image-based in situ transcriptomics has not been specifically addressed by most mapping models. Besides, since ST is a newly emerging technology, only a few models are applied in this field and its efficacy needs to be developed [10]. Deconvoluted tissues are limited in the spatially resolved at the original scale of ST arrays and ignore the adjacent tissues [31]. With the developments of SRT, more and more integrating models may be explored to face the challenges of mapping and deconvolution.

## Application of integration in TME

Recent work in combining scRNA-seq with spatial technologies has focused on tissue homeostasis, tissue development, disease, and tumor microenvironment. The integration of two approaches may break the bottlenecks in the spatial resolution of neighboring cell subpopulations in cancer, and help to describe the signaling circuitry about the intercommunication and its exact mechanisms in producing different types and malignant stages of tumors. The TME is the most widely used area in SRT, and it is closely related to cancer malignant behaviors, which are governed by crosstalk within and across all cellular compartments [32–35]. In addition, a modern (though not exhaustive) list of key molecular markers of cell populations discussed below in the TME with their characteristics is given in Table 1. A deeper study of intricate spatial patterning of single cells brings biological insight into cellular and spatial heterogeneity between and within tumors in a complex environment. Moreover, it also has the potential to explain how TME affects cellular infiltration and interaction, which represent an attractive target for treatment and prognosis.

**Table 1:** Key molecular markers of each cell type in the TME

Population	Subtypes	Marker	References
CAFs	–	α-SMA, vimentin, FAP, PDPN, PDGFRα/β, FSP1, DDR2, S100A4, CD10, GPR77	[36–41]
MSCs	–	CD105^+^, CD73^+^, CD70^+^, CD13^+^, CD29^+^, CD44^+^, CD10^+^, CD45^−^, CD34^−^, CD14^−^ or CD11b^−^, CD79a^−^, HLA-DR^−^	[42–44]
TAMs	M1	CD68, CD11b, CD80, CD86	[45, 46]
	M2	CD68, CD11b, CD163, CD206	
DCs	cDC1	XCR1, CD45, CADM1, CLEC9A, CD141	[47, 48]
	cDC2	CD45, CD1C, FcεR1A, CD172A	
	pDCs	CD45RA, CD123, CD2	
Endothelial cells	–	PECAM1, CD31, CD34, CD13, CD29	[49, 50]
CD4^+^ T cells	–	CD3^+^CD4^+^CD8^−^	[51–54]
	Th1 cells	CXCR3	
	Th2 cells	CCR4	
	Th17 cells	CCR6	
	Th22 cells	CCR10	
	T_reg_ cells	CD4^+^CD25^+^Foxp3^+^	
CD8^+^ T cells	-	CD3^+^ CD8^+^CD4^−^	
	Tc1 cells	CRCX3, IRF4	
	Tc2 cells	CCR4, CRTH2, GATA3	
	Tc9 cells	CRCX3, IRF4, IL9, IL10	
	Tc17 cells	CCR6, IL23R, IRF4, IL17	
MDSCs	PMN-MDSCs	CD11b^+^CD33^+^HLA^−^DR^−^/CD14^−^CD15^+^	[55, 56]
	M-MDSCs	CD11b^+^CD33^+^HLA^−^DR^−^/CD14^+^CD15^−^	

*CAFs* cancer-associated fibroblasts, *MSCs* mesenchymal stem cells, *TAMs* tumor-associated macrophages, *DCs* dendritic cells, *cDCs* conventional DCs, *pDCs* plasmacytoid DCs, *MDSCs* myeloid-derived suppressor cells, *PMN-MDSCs* granulocyte-like MDSCs, *M*-*MDSCs* monocytic MDSCs

**Fig. 2:**
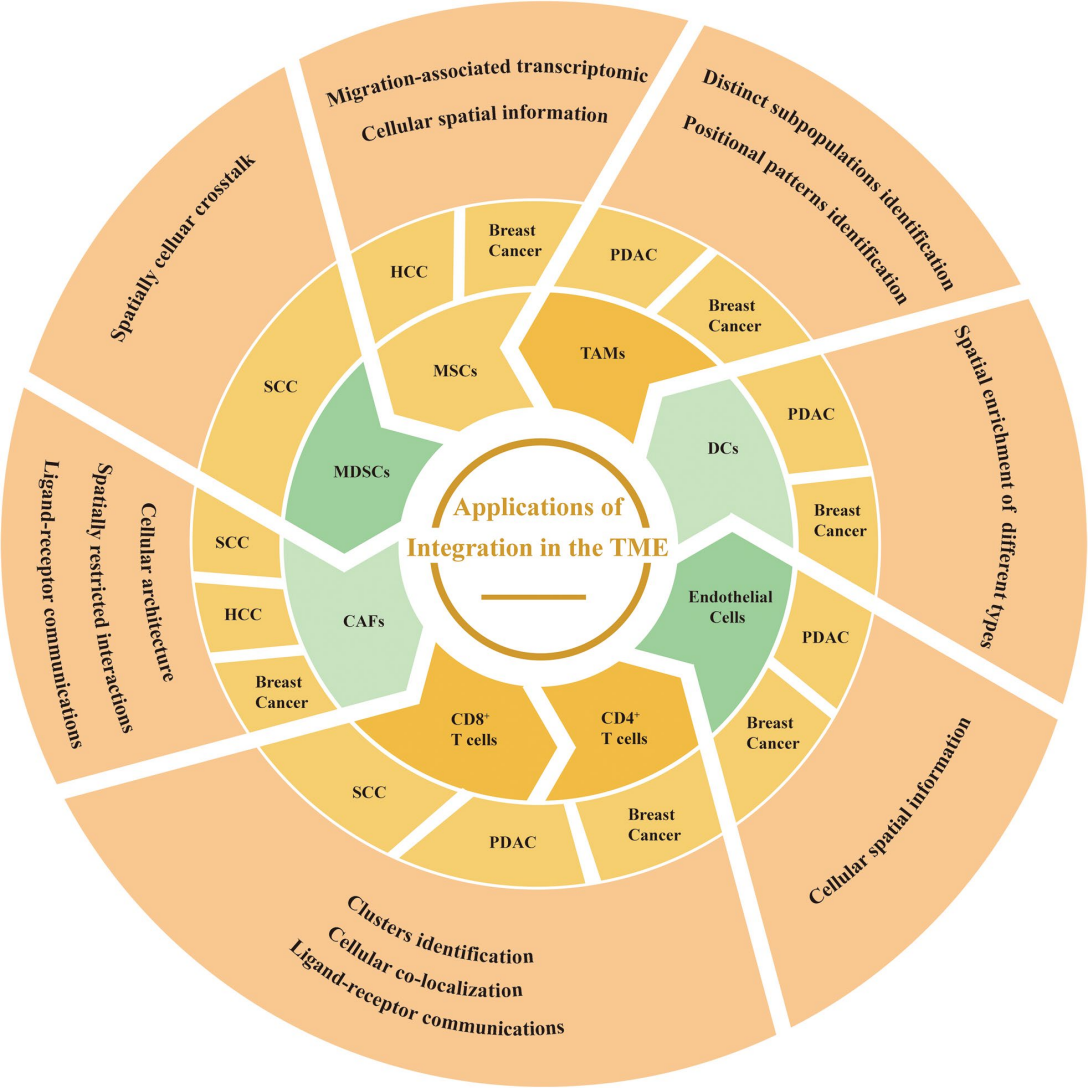
A schematic overview: application of integration for cell types in the TME. CAFs: cancer-associated fibroblasts; MDSCs: myeloid-derived suppressor cells; MSCs: mesenchymal stem cells; TAMs: tumor-associated macrophages; DCs: dendritic cells; SCC: squamous cell carcinoma; HCC: hepatocellular carcinoma; PDAC: pancreatic ductal adenocarcinoma

Here, we show applications of integrating scRNA-seq with SRT in cellular components in TME. Combining datasets of scRNA-seq and ST could map signaling between adjacent tumor and TME cells at the leading edge, suggesting advances in mapping cellular crosstalk at leading-edge niches [35]. Furthermore, we give a schematic overview of the applications of integration methods on the different populations present within the microenvironment (Fig. 2)*.*

## Stromal cells

TME is composed of tumor cells and nearby endogenous stromal cells [57, 58]. Stromal cells recruited from the local host stroma range in types and include CAFs (cancer-associated fibroblasts) and MSCs (mesenchymal stem cells) that promote extracellular matrix remodeling, cellular migration, angiogenesis, and evasion of immunosurveillance in tumor growth and development [58, 59]. Reactive stroma can be regarded as an emerging hallmark of cancer initiation and progression. Berglund et al. revealed an unexplored landscape of heterogeneity through spatial maps of prostate cancer. It enabled de-novo characterization and delineation of reactive stroma in the proximity of cancer and inflammation, uncovering high levels of oxidative stress and ILK signaling within the reactive stroma. It indicated that cancer depended on stroma to release energy to support tumor growth and survival [60].

## Cancer-associated fibroblasts

CAFs are one of the dominant entities in the stroma of many cancers, including breast cancer, hepatic cell carcinoma, human squamous cell carcinoma, and lung cancers. CAFs are a heterogeneous population of irreversibly activated fibroblasts that serve distinct, critical functions in tumor metabolism, immunity, drug resistance, negative regulation, tumorigenesis, and metastasis [36, 61–63]. Besides, numerous studies have shown the metastasis potential of cancer cells depending on CAFs, like in lung cancer, squamous cell carcinoma lung metastasis, and colorectal cancer [38, 64–66]*.* It has distinct subsets of functional fibroblasts differentiated from resting fibroblasts, such as tumor-restraining (F1), tumor-promoting (F2), secretory (F3), and ECM-remodelling (F4) subtypes detected and identified by various means based on the expression of a limited set of cell surface markers, such as α-SMA, vimentin, FAP, PDPN, PDGFRα/β, FSP1, DDR2, and S100A4 [36–39]. Recently, some studies have identified CD10 and GPR77 as specific fibroblast surface markers which facilitate live-cell sorting of CAFs in breast cancer [40, 41].

In breast cancer, four subpopulations of CAFs, such as vascular CAFs, matrix CAFs, cycling CAFs, and developmental CAFs, and their distinct gene programs were revealed by scRNA-seq with high resolution [67]. A single-cell and spatially resolved atlas of human breast cancers described the cellular architecture and spatially restricted interactions with the immune system. Stereoscope belongs to deconvolution as a strategy of combining scRNA-seq and ST, of which two datasets came from different breast cancer tissue samples. Stereoscope identified spatially distinct subpopulations of CAFs, with myofibroblast-like enriched in invasive cancer regions, while inflammatory CAFs were scattered in invasive cancer, stroma, and TIL-aggregate regions in different clinical subtype samples. In addition, this study also explored the top ligand-receptor interactions between most CAFs and CD4^+^/CD8^+^ T cells, suggesting that CAFs may directly regulate immune cells [49]. Moreover, inflammatory CAFs were enriched in the stress-response region of pancreatic ductal adenocarcinoma (PDAC) [32].

As for the human liver microenvironment, this study applied scRNA-seq, LCM-seq, and smFISH in cholangiocarcinoma, colorectal liver metastasis, and benign tissue samples. A developed approach named AutoGeneS deconvoluted scRNA-seq with transcriptomics of LCM tissue to obtain zonation patterns of human hepatocytes when prior knowledge of landmark genes was lacking [68, 69]. This study revealed the far distance between CAFs and endothelial cells, and the interactions between CAFs and scar-associated macrophages (SAMs). CAFs, which were most abundant in the fibrotic zones, produced most of the collagen and lamina proteins, interacting with integrin receptors on tumor cells [70].

In a study of human squamous cell carcinoma, CAFs were modulated by an immunosuppressive tumor-specific keratinocyte subpopulation that expressed immunotherapy resistance genes in a fibrovascular niche at the tumor borders through ligand-receptor communications [10, 35]. By using the deconvolution strategy, this study indicated the contribution of CAFs to tumor progression, immunosuppression, and heterogeneity.

Thus, researchers propose a cellular, molecular, and spatial functional taxonomy of CAFs by using the integration of scRNA-seq and ST, opening up the possibility for the development of novel targeted drugs aiming to block CAFs-immune or CAFs-tumor cell signaling [49, 67, 70].

## Mesenchymal stem cells

MSCs are multipotent stromal cells that can differentiate into cells of the mesodermal lineage providing structural support to organs, synthesizing and remodeling the ECM, and regulating development [71]. MSCs play an important role in tumor development at various stages of progression, which modifies several effector functions [72, 73]. MSCs generally express CD105, CD73, CD70, CD13, CD29, CD44, and CD10, and lack expression of CD45, CD34, CD14 or CD11b, CD79a or CD19, and HLA-DR [42–44]. Recent studies have found that MSCs may differentiate at the site of the tumor and interact with tumor cells through paracrine signaling. Cross-talk between tumor cells and MSCs has been shown to increase metastatic potential. In colorectal cancer, MSCs have been found to increase tumor migration and invasion through IL-6/JAK2/STAT3 signaling, providing a novel therapeutic or preventive target [74].

Lee et al. used XYZeq in hepatocellular carcinoma (HCC) and found that MSCs have differentially expressed genes regulating ECM. It suggested that hepatocellular carcinoma cells may induce a local gene expression program in MSCs nearby that could contribute to malignant remodeling of the ECM. The location of the tumor cells and non-tumor cells may determine heterogeneous gene expression in MSCs. Transcriptionally variable genes within MSCs were driven by their location within the complex tissue architecture [26]. Observations in breast cancer showed MSCs were often spatially segregated underscoring the role of TME in their differentiation and migration. These findings suggested it was possible to block stromal signaling or differentiation as therapeutic strategies [49]. It has an advantage for joint analysis of spatial and single-cell transcriptomic to reveal not only local information but also migration-associated transcriptomic programs in MSCs.

## Tumor-associated macrophages (TAMs)

Many supporting cells having distinct functions during tumorigenesis are derived particularly from the myeloid lineage especially TAMs (tumor-associated macrophages) [4, 75]. TAMs polarize into two functional phenotypes: the M1 state (pro-inflammatory and anti-tumor) and the M2 state (anti-inflammatory and pro-tumor) [34]. The distinct two phenotypes may influence cancer progression and overall survival [76]. CD68 and CD11b are co-markers for M1 and M2 macrophages [45]. CD80, CD86 are specific for M1 subtypes, while CD163, CD206 are specific for M2 subtypes [45, 46]. The existing problems with TAMs are that this classification is oversimplified because it does not fully represent the complexity of macrophage activation and its positional patterns [4, 77]. Macrophages have a leading position in pathophysiological responses, such as TME, which paves the way to tumorigenesis [78, 79]. In progressive cancer, TAMs recruited to TME are fast becoming a key instrument in cancer cell proliferation, immunosuppression, and angiogenesis in support of tumor growth and metastasis [79, 80]. In colorectal cancer, Yu et al. have found that TAMs play a key role in cancer proliferation depending on MMP1 via accelerating cell cycle transition [81]. Wei et al. have shown the crosstalk between TAMs and colorectal cancer cells which is associated with cancer migration, invasion, and circulating tumor cell-mediated metastasis [82]. Dora et al. have characterized TAMs in neuroendocrine-high and -low small cell lung cancer [76]. Furthermore, spatial density and distribution and gene expression of TAM phenotypes have been shown prognostic value in non-small-cell lung carcinoma (NSCLC) [83].

10 × Chromium (scRNA-seq), 10 × Genomics (ST), and their deconvolution method were used to identify two large TREM2-high lipid-associated macrophages (LAMs) that were similar to the PD-L1^+^ macrophage population associated with high clinical grade and exhausted T cells in breast cancers [49, 84]. Additionally, LAMs and CXCL10^hi^ macrophages were relevant to immunosuppression and were paratactic to PD-1^+^ lymphocytes. As TAMs were associated with poor prognosis and are emerging targets for cancer immunotherapy, nine ecotypes driven by cells spanning the major lineages in primary breast cancers were defined. The cellular composition and tumor biology of each ecotype were similar [49, 84–86].

Elosua-Bayes et al. developed SPOTlight, a deconvolution that enabled the integration of ST with scRNA-seq data in PDAC publicly available reference, finding a remarkable enrichment in the tumor region of pro-inflammatory M1 TAMs while anti-inflammatory M2 TAMs were enriched in normal pancreas tissue, endothelial, and endocrine cells in different tissue regions [87]. In another PDAC study, M2 TAMs were most enriched in the ducts, while inflammatory M1 TAMs expressing IL1B were more enriched in the stroma and cancer regions, which was consistent with Bayes’s study [32, 88]. It illustrated opposite positional patterns of enrichment in two subtypes of macrophages through MIA. The multimodal intersection analysis (MIA) approach integrated scRNA-seq and spatially barcoded oligo-deoxythymidine microarrays. Moncada et al. used two melanoma ST samples to validate MIA, in which macrophages were restricted to the melanoma region periphery or a particular region within the larger annotated melanoma area, indicating that macrophages were in spatially restricted regions in melanoma [32].

Therefore, a detailed subpopulation of TAMs with expression levels for genes and their specific relationships in positional patterns can be revealed by integrating methods, which may have the potential to refine classification.

## Dendritic cells

Dendritic cells (DCs) are potent antigen-presenting cells, which can present antigen to T cells and activate these cells to enhance the immune response [89]. Previous studies have distinguished two types of DCs: one is conventional DCs (cDCs), while another is plasmacytoid DCs (pDCs) [90]. Human cell surface markers of pDCs are CD45RA, CD123, and CD2 [47]. Besides, cDCs have two subsets, cDC1 and cDC2, with distinct cell surface markers and functions [48]. cDC1 are generally defined by XCR1, CD45, CADM1, CLEC9A, and CD141, while cDC2 are described by CD45, CD1C, FcεR1A, CD172A [47]. DCs are a promising therapy in cancer treatment. In early-stage PDAC, overcoming cDCs deficiency has led to disease restraint. Otherwise, in advanced PDAC, restoration of cDC function has led to restoring tumor-restraining immunity [91]. In addition, functional DCs in tumor regions were excluded from lung cancers dynamically, which may support malignant progression [92].

In transcriptional profiling of human breast cancer, Wu et al. identified three types of DCs, cDCs, pCDs, and LAMP3 high DC population, which was not previously detected in single-cell studies of breast cancer [49]. In PDAC via high throughput single-cell sequencing and MIA, two subpopulations of DCs, A and B, were identified. Subpopulation A was enriched in pancreatic tissue, while subpopulation B was enriched in the ducts of the tissue [32].

## Endothelial cells

Tumor endothelial cells play a critical role in cancer cell metastasis and dormancy exhibiting unique phenotypic and functional characteristics when compared to normal endothelial cells [93]. Many studies have shown that the proliferation and motility of tumor endothelial cells are associated with several pathological processes for tumor progression and metastasis, such as microvessel sprout formation and angiogenesis [94]. PECAM1, CD13, CD29, CD31 and CD 34 are main markers for endothelial identification [49, 50]. In breast cancer, Ma et al. have identified the heterogeneity of endothelial cells and indicated its potential role in contributing to cancer metastasis [95]. Besides, lung cancer cells have been shown to promote endothelial cell tube formation which changes the TME to facilitate tumor growth [94]. In colorectal cancer, TME-dependent heterogeneity of tumor endothelial cells regulated by SPARCL1 has promoted tumor cell dormancy and vessel homeostasis [96]. Furthermore, Meng et al. have shown the role and mechanism of Hsp90β in tumor endothelial cell-dependent angiogenesis and its therapeutic value in hepatocellular carcinoma [97].

Three endothelial states (s1, s2, and s3) were identified through a single-cell and spatially resolved atlas of human breast cancers. These three states mainly in the normal, stroma, and lymphocytes areas were dynamic and interconvertible, suggesting that these endothelial cells may serve as resident cell types in the TME [49]. Otherwise, in one PDAC sample, endothelial cells were significantly enriched in the interstitium using integration of two advanced techniques [32]. These insights, such as revealing spatial information of different subpopulations of endothelial cells, may provide a deeper understanding of cancer metastasis and dormancy.

## CD4^+^ and CD8^+^ T cells

Tumor-infiltrating lymphocytes (TIL) play a crucial role in TME, which is associated with cancer progression, response to therapy, and clinical outcomes [98]. In addition, studies of TIL mainly focus on T cells. T cell infiltration formed in human cancer is a regulator of natural disease progression and also determines the probability of clinical response to cancer immunotherapy, which may provide potential prognostic value [99]. There are three main types of T cells: helper T cells (T_H_ cells/CD4^+^ T cells), cytotoxic T cells (T_C_ cells/CD8^+^ T cells), as well as regulatory T cells (T_reg_ cells). CD4^+^ T cells generally express CD3^+^CD4^+^CD8^−^, while CD8^+^ T cells generally express CD3^+^ CD8^+^CD4^−^ [53]. CD4^+^CD25^+^Foxp3^+^ are used to describe T_reg_ cells [51, 52]. In addition, different subsets of CD4^+^ and CD8^+^ T cells have their specific markers, such as Th1 cells (CXCR3), Th2 cells (CCR4), Th17 cells (CCR6), Th22 cells (CCR10), Tc1 cells (CRCX3 and IRF4), Tc2 cells (CCR4, CRTH2, and GATA3), Tc9 cells (CRCX3, IRF4, IL9, and IL10), Tc17 cells (CCR6, IL23R, IRF4, and IL17) [53, 54]. CD8^+^ T cells encounter dysfunction and exhaustion due to immunosuppression within the TME during tumor development and progression [100]. CD4^+^ T cells play a key role in the adapted immune system, which can variously target tumors either directly by eliminating tumor cells through cytolytic mechanisms or indirectly by modulating TME [101–103]. Hiraoka et al. have indicated that deeper infiltration by both CD8^+^ and CD4^+^ T cells presents a better prognosis for patients with NSCLC [104]. Another study has mapped the heterogeneity of TILs in NSCLC, which may attribute to cancer immunotherapy [105]. Yang et al. have shown the association between CD8^+^ and CD4^+^ T cell-related genes and colon cancer prognosis [106]. In addition, high infiltration of lymphocytes has been observed in one subpopulation characterized by low peroxisome and high TIM3 of colorectal cancer [107].

SPOTlight applied to PDAC samples annotated 12 T cells and predicted the proportion within each capture spot. Recently activated CD4^+^, pre-exhausted CD8^+^, and proliferative CD8^+^ T cells significantly increased in tumor regions, while most transitional memory CD4^+^ T cells were in normal tissue. Intriguingly, recently activated CD4^+^ T cells co-localized with pre-exhausted CD8^+^ T cells in tumor areas and could not be detected through their presence alone, indicating a possible target for precise pathology assessments [87]. In breast tumor samples, 18 T-cell and innate lymphoid clusters were identified. One subset of exhausted CD8^+^ T cells named *LAG3/c8* in triple-negative breast cancer (TNBC) had higher expression of PD-1, LAG3, and the ligand-receptor pair of CD27 and CD70, known to enhance T cell cytotoxicity [49, 108]. In human squamous cell carcinoma, CD8^+^ T cells were observed to co-localize with T_reg_ cells in the compartmentalized tumor stroma, which showed a feature of potential immunosuppression [35]. Such visualization underlined interactions between T cells that mediate the tumor immune environment and can shed new light on the peculiarities of tumor microenvironments [10, 87].

## Myeloid-derived suppressor cells

Myeloid-derived suppressor cells (MDSCs) are a heterogeneous population of immature myeloid cells with immunosuppressive functions. When in pathological conditions, especially cancer, the differentiation, and maturation of immature myeloid are stopped leading to the expansion of MDSCs in vivo [109]. MDSCs consist of two large groups of cells: granulocyte-like MDSCs (PMN-MDSC or G-MDSC) and monocytic MDSCs (M-MDSCs) [110, 111]. PMN-MDSCs can be described as CD11b^+^CD33^+^HLA^−^DR^−^/CD14^−^CD15^+^, while M-MDSCs can be defined as CD11b^+^CD33^+^HLA^−^DR^−^/CD14^+^CD15^−^ in human [55, 56]. MDSCs play an important role in immune surveillance in TME via immunosuppressive mechanisms, such as metabolic mechanisms, STAT signaling pathway, and endoplasmic reticulum stress in lung cancers [112]. Besides, high levels of MDSCs have been associated with resistance to several therapeutic strategies, like chemotherapy and immunotherapy with a poor prognosis [112]. Huang et al. have found modulating MDSCs in TME may improve the efficacy of EZH2 inhibitors to suppress antitumor immunity [113]. Another study also showed the potential therapeutic target of PMN-MDSC to overcome resistance to immune checkpoint inhibition in NSCLC [114]. In colorectal cancer, reprogramming MDSCs may have the potential to enhance the efficacy of therapeutic strategies [115].

Multimodel profiling of cutaneous squamous cell carcinoma, MDSCs were identified and highly expressed several potential mediators of T_reg_ recruitment, such as CCXL9/10/11, CCL4, and CCL20, via the integration of scRNA-seq and ST [35]. In addition, extensive autocrine and paracrine interactions between MDSCs and tumor-specific keratinocytes revealed cellular crosstalk at leading-edge niches [35]. However, most studies have just identified myeloid cells without identifying MDSCs when using scRNA-seq and ST techniques. MDSCs have emerged as an important contributor to tumor progression, so it’s quite important to reveal the spatial information and cellular interactions of MDSCs, which may benefit cancer therapeutic strategies.

## Conclusions

Integrating scRNA-seq with SRT is beneficial in understanding cell-type proportions to the proximity of tissue architecture. It also helps to study intercellular communications through expressions of ligands and receptors in TME, which may be beneficial to define disease subtypes, provide potential therapeutic targets, and predict prognosis. Additionally, integrating methods can be used to describe the atlas at the single-cell resolution of healthy or diseased tissues and explore normal tissue homeostasis and tissue ontogeny at key points. Nowadays, SRT is growing at a rapid pace with improvements in resolution, sensitivity, throughput, as well as accessibility. Despite deconvolution and mapping algorithms, new learning algorithms are exploited to define the most relevant features of biological function in SRT. However, it’s costly when applying scRNA-seq and SRT using matched samples or publicly available references. Considering the original scale of the ST technology, deconvolved mixtures are still only spatially resolved and the proximity structure of cell types cannot be recovered. Recently, real-time cell tracking based on SRT at single-cell resolution has been developed to monitor spatially resolved intercellular tissue dynamics in real-time elucidating metastatic progression and immune cell dynamics in disease, which has an extensive prospect in cancer research.

## Data Availability

Not applicable.

## Abbreviations

TME: Tumor microenvironment
ECM: Extracellular matrix
RNA-seq: RNA-sequencing
NGS: Next-generation sequencing
bulk RNA-seq: Bulk RNA-sequencing
scRNA-seq: Single-cell RNA sequencing
SRT: Spatially resolved transcriptomics
ISH: In situ hybridization
ISS: In situ sequencing
LCM: Laser capture microdissection
LCM-seq: LCM sequencing
smFISH: Single-molecule RNA fluorescence in situ hybridization
MERFISH: Multiplexed error-robust fluorescence in situ hybridization
seqFISH: Sequential fluorescence in situ hybridization
osmFISH: Ouroboros smFISH
FISSEQ: Fluorescent in site RNA sequencing
STARmap: Spatially resolved transcript amplicon readout mapping
SNV: Single nucleotide variants
ST: Spatial transcriptomics
UMI: Unique molecular identifier
CAFs: Cancer-associated fibroblasts
MSCs: Mesenchymal stem cells
HCC: Hepatocellular carcinoma
PDAC: Pancreatic ductal adenocarcinoma
SAM: Scar-associated macrophages
TAMs: Tumor-associated macrophages
LAMs: Lipid-associated macrophages
NSCLC: Non-small-cell lung carcinoma
MIA: Multimodal intersection analysis
DCs: Dendritic cells
cDCs: Conventional DCs
pDCs: Plasmacytoid DCs
TIL: Tumor-infiltrating lymphocytes
T_H_ cells: Helper T cells
T_C_ cells: Cytotoxic T cells
Treg cells: Regulatory T cells
TNBC: Triple-negative breast cancer
MDSCs: Myeloid-derived suppressor cells
PMN-MDSCs: Granulocyte-like MDSCs
G-MDSCs: Granulocyte-like MDSCs
M-MDSCs: Monocytic MDSCs
